# Quantum chemical calculations, spectroscopic studies and molecular docking investigations of the anti-cancer drug quercitrin with B-RAF inhibitor

**DOI:** 10.1016/j.heliyon.2022.e09539

**Published:** 2022-05-26

**Authors:** M. Govindammal, S. Kannan, P. Srinivasan, M. Prasath

**Affiliations:** a Department of Physics, Periyar University Centre for Post Graduate and Research Studies, Dharmapuri, 635 205, India; b Centre for High Computing, CSIR-Central Leather Research Institute (CLRI), Chennai, 600020, India; c Chikkaiah Naicker College, Erode, 638004, India

**Keywords:** Lung cancer, B-RAF kinase, DFT

## Abstract

Quercitrin is an anti-lung cancer agent. It is a naturally occurring flavonoid and its derivatives are mainly present in nuts and beverages. It is mainly available as a glycoside, and the quercitrin glycosides are found to prevent the metastasis of cancer. Quercitrin is optimized with 6–311++G(d,p) basis set using the B3LYP method to attain its minimum energy structure. The vibrational studies of the Quercitrin compound were elucidated with reference to Potential Energy Distribution (PED). The geometrical parameters were obtained and correlated with experimental values. To examine the nature of the charge transfer mechanism of Quercitrin, the HOMO-LUMO energy gap is computed. The anti-cancer activity of Quercitrin has been explored using molecular docking study that are used to estimate how the ligand interacts with protein, specifically to identify the best-fit orientation of the ligand, its binding mode, and intermolecular interactions of amino acid residues in the binding region of B-RAF kinase protein. The binding affinity of the compound Quercitrin (-7.14 kcal/mol) was found using AutoDock and validated with a Glide XP score in Schrodinger tool (-8.01 kcal/mol). MD simulations of protein-ligand complexes were monitored for 100 ns, from which the RMSD, RMSF, Rg, H-bonds, and interaction energy calculations were executed. From these investigations, it is identified that the compound quercitrin has maintained good structural stability, compactness, higher Hydrogen bonds, and interaction energies than the Imidazopyridinyl benzamide inhibitor.

## 1 Introduction

Lung cancer is the crucial reason for morbidity and mortality worldwide in both sexes and is most widely recognized as a reason for malignancy-related deaths [1]. Non-Small Cell Lung cancer (NSCLC) is a highly aggressive and recurrent kind of lung cancer. Globally, NSCLC is considered responsible for about 85% of all lung cancers [2]. B-RAF is a kinase protein and a member of the RAF family of Ser/Thr kinase family which is highly downstream of the Ras/Raf/MEK/ERK signaling pathway [3, 4]. B-RAF mutations have been found in 3–4% of NSCLC [5]. If the MEK/ERK signaling pathway is activated in human cancer, B-RAF kinases are supposed to be a prominent therapeutic target. B-RAF is involved in complex activity such as phosphorylation/dephosphorylation process resulting in kinase activation/deactivation [6, 7].

Quercitrin is an active natural bioflavonoid, a plant derivative that mostly appears in the glycoside structure [8]. The bounded sugar moiety in the Quercitrin compound makes the Quercitrin aglycone soluble and improves the absorption and bioavailability. Quercitrin exhibits anti-cancer properties against NSCLC. It is reported to inhibit quercitrin–induced apoptosis in the NSCLC cell line A549 with an IC_50_ value of 50 μm [9]. Quercitrin displays an anti-oxidant property and has become the focus point for revealing the anti-cancer activity [10]. Vibrational spectroscopic studies have been carried out to recognize and examine the chemical bonding and intramolecular bonds of quercitrin. The vibrational analysis of Quercitrin was discovered using quantum chemical calculations using the DFT approach. The theoretical findings match up with the experimental results. The most reactive area of the Quercitrin is illustrated using the molecular electrostatic potential map (MEP). The chemical reactivity of quercitrin is determined by the Fukui function. The bio-availability compound was scrutinized with ADMET prediction analysis along with Lipinski's rule of five. A molecular docking evaluation was employed to explore the intermolecular interactions of quercitrin in the binding site of the B-RAF kinase protein. The analyses, such as HOMO-LUMO, MEP, and ADMET predictions, are utilized to find the anti-cancer behavior of Quercitrin against NSCLC.

## 2 Materials and methods

### 2.1 Experimental methods

Quercitrin was procured in solid form with a purity of ≥95% and no further purification of the compound has been made for spectral studies. The PERKIN ELMER (4000-400 cm^−1^) spectrometer with the KBr pellet technique was used to obtain the FT-IR spectrum of Quercitrin. Additionally, the FT-Raman spectrum was accomplished for Quercitrin in the region of 4000–100 cm^−1^ with a resolution of 2 cm^−1^ employing Brucker RFS 27- Nd: YAG laser source of 1064 nm at SAIF-IITM.

### 2.2 Computational methods

The Quercitrin compound was optimized using the DFT method employing the B3LYP level of theory by *Gaussian09* software [11]. The geometrical parameters of the optimized geometry were generated using the *Gaussview 0.5* software package [12]. The *VEDA 4xx* program package [13] was used to obtain the Potential Energy Distribution percentage (PED %) and fundamental vibrational assignments of Quercitrin. The biological properties of the ADME assessment of the compound were obtained using an online tool, the *Molinspiration Cheminformatics server*. The ligand (Quercitrin) and protein (B-RAF kinase) were docked with the aid of *Autodock 4.2* [14]. The structural and interaction patterns of the protein-ligand complex are visualized and generated using the *PyMol* [15], *Chimera* [16], and *Ligplot* [17] software packages. The genetic algorithm and Lamarckian GA were employed to search for the appropriate conformers and for binding affinity. The number of points in the X, Y and Z dimensions are 24, 26, and 24 respectively. The center grid box in the X, Y and Z centres are -23.905, 5.081, and -6.037 respectively. The total grid points per map are 16875, with a spacing of 1 Å. The docking conformations are generated in AutoDock Vina, and those conformations are graded on the basis of binding free energy, by applying Eq. (1),

(1) ΔG_bind_ = ΔG_vdw_ + ΔG_desolv_ + ΔG_Hbond_ + ΔG_ele_ + ΔG_internal_ + ΔG_torsion_ - ΔG_unbound_

where ΔG_bind_ represents the binding free energy, ΔG_vdw_ the van der Waals interaction, ΔG_desolv_ the desolvation effect, ΔG_Hbond_ the hydrogen bonding interaction, ΔG_ele_ the electrostatic interaction, ΔG_internal_ the internal energy, ΔG_torsion_ and ΔG_unbound_ represents the torsional free energy, and unbound systems energy respectively.

The *AutoDock* results were further validated with the Extra Precision Glide XP score in *Maestro*, the *Schrodinger suite* [18]. The molecular dynamics (MD) simulation of docked structures of protein-ligand complexes was carried out for a 100 ns time period using the *GROMACS* program, version 2019.5 [19]. With the SPC216 (simple point charge) water model, the molecular system was solvated in a cubic box [20]. *Amber 99SB* force field was executed and the system was examined at the same temperature of 300 K over a 100 ns period of time. The PBC (periodic boundary condition) was applied to the system and the ligand topology parameters were generated using the *ACPYPE* server [21]. The system was energy minimized and the position restrained MD was carried out for 1000 ps using NVT and NPT ensembles with the Parrinello-Rahman pressure coupling [22]. The RMSD, RMSF, radius of gyration and interaction energy were analyzed using the *GROMACS* program.

## 3 Results and discussion

### 3.1 Structural aspects

**Figure 1:**
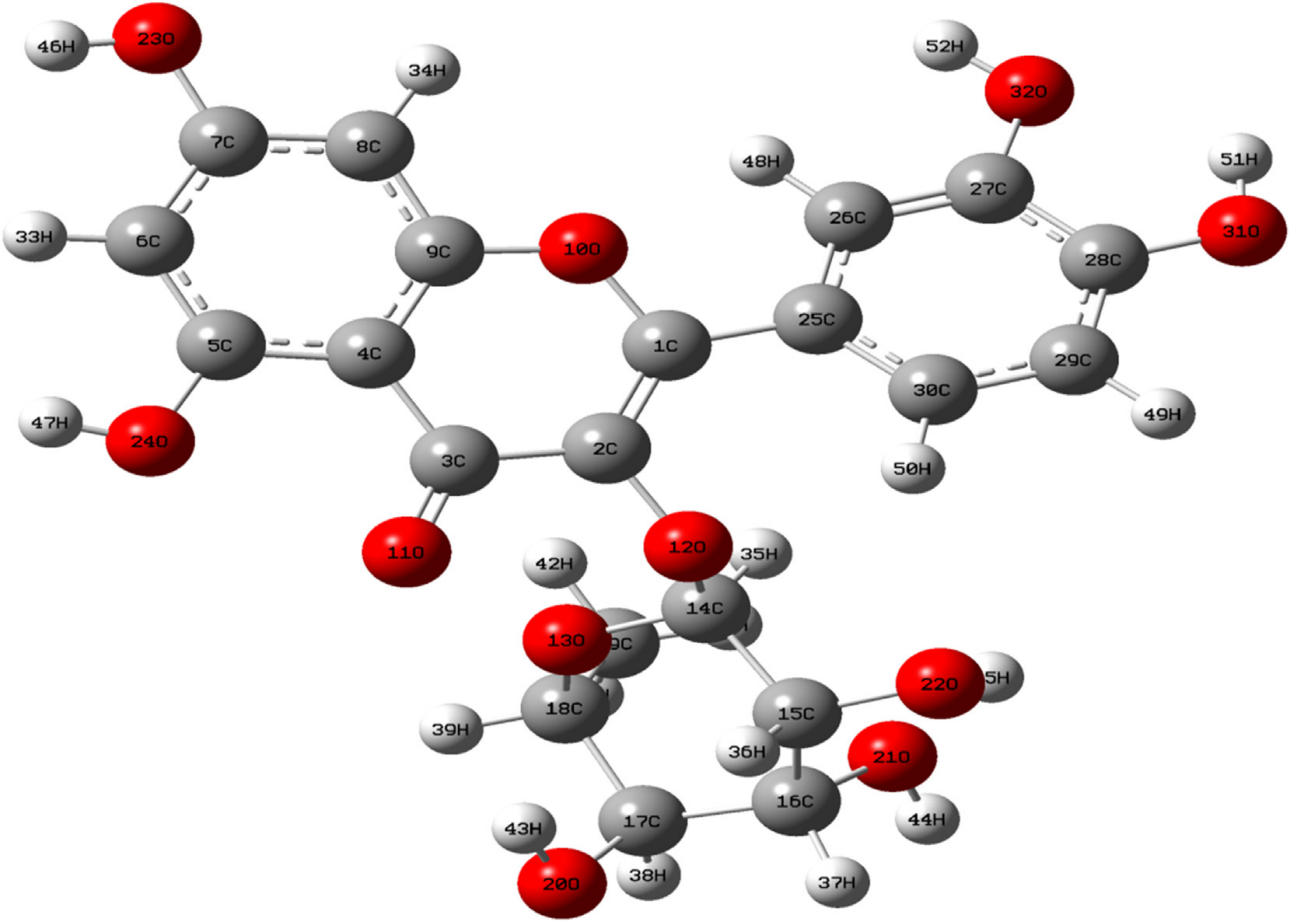
The scaled ball and stick model of optimized structure of Quercitrin molecule with atoms numbering scheme.

The Quercitrin structure was drawn utilizing the *Chemdraw8.0 tool* [23] and optimized with the DFT method to get the energy minimized structures using the B3LYP levels of theory [24, 25]. The Optimized geometrical parameters of the Quercitrin compound were generated using Gaussian 09 and GaussView5.0 as shown in Figure 1. With the crystal structure of Quercitrin not yet known, we have calculated the theoretical predictions on structural parameters of Quercitrin with available X-ray diffraction data [26] and the results are presented in Table 1. The Quercitrin's threshold converged at the limits of its maximum force value of 0.000010 (a.u) and displacement value of 0.000878 (a.u). The dipole moment value of the compound is found to be 8.2324 Debye. The quercitrin contains twelve C–H, twenty C–C, fifteen C–O, and eight O–H bonds. The C–C bond lengths of the phenol ring range from 1.359 to 1.369 *Å*. The ketone groups in Quercitrin have bond lengths of C(2)–O(12)→1.376/1.384∗*Å*, C(3)–O(11)→1.218/1.266∗*Å*, C(9)–O(10)→1.363/1.373∗*Å*, and O(1)–O(10)→1.366/1.373∗ (∗ denotes the Observed value). The highest bond length value, found in Quercitrin is C(16)–C(17) with 1.542 *Å* due to a double bond. The ketone group exhibits the bond angle of C(2)–C(1)–O(10) and the value is found to be 119.9/120.5°∗.

**Table 1:** Bond lengths and bond angles of quercitrin molecule compared with XRD data.

Bond Length (Å)	Calculated	Exp^a^	Bond Angle (^o^)	Calculated	Exp^a^
C(1)-C(2)	1.359	1.369	C(2)-C(1)-O(10)	119.9	120.5
C(1)-O(10)	1.366	1.373	C(2)-C(1)-C(25)	128.9	127.8
C(1)-C(25)	1.473	1.468	O(10)-C(1)-C(25)	111.1	111.6
C(2)-C(3)	1.480	1.445	C(1)-C(2)-C(3)	122.9	120.9
C(2)-O(12)	1.376	1.384	C(1)-C(2)-O(12)	121.1	122.1
C(3)-C(4)	1.479	1.433	C(3)-C(2)-O(12)	115.8	116.7
C(3)-O(11)	1.218	1.266	C(2)-C(3)-C(4)	113.7	116.2
C(4)-C(5)	1.419	1.429	C(2)-C(3)-O(11)	121.5	121.6
C(4)-C(9)	1.404	1.402	C(4)-C(3)-O(11)	124.8	122.2
C(5)-C(6)	1.393	1.359	C(3)-C(4)-C(5)	123.9	122.4
C(5)-O(24)	1.351	1.359	C(3)-C(4)-C(9)	119.6	120.6
C(6)-C(7)	1.400	1.399	C(5)-C(4)-C(9)	116.5	116.9
C(6)-H(33)	1.087	1.393	C(4)-C(5)-C(6)	120.6	120.9
C(7)-C(8)	1.387	-	C(4)-C(5)-O(24)	118.5	118.8
C(7)-O(23)	1.361	1.347	C(6)-C(5)-O(25)	120.8	120.3
C(8)-C(9)	1.392	1.373	C(5)-C(6)-C(7)	120.5	119.9
C(8)-H(34)	1.081	-	C(5)-C(6)-H(33)	119.5	-
C(9)-O(10)	1.363	1.373	C(7)-C(6)-H(33)	120.0	-
O(12)-C(14)	1.410	1.442	C(6)-C(7)-C(8)	120.5	121.2
O(12)-H(50)	2.289	-	C(6)-C(7)-O(23)	122.0	116.2
O(13)-C(14)	1.420	1.398	C(8)-C(7)-O(23)	117.5	122.5
O(13)-C(18)	1.440	1.442	C(7)-C(8)-C(9)	118.2	118
C(14)-C(15)	1.535	1.517	C(7)-C(8)-H(34)	121.0	-
C(14)-H(35)	1.098	-	C(9)-C(8)-H(34)	120.8	-
C(15)-C(16)	1.536	1.534	C(4)-C(9)-C(8)	123.7	123
C(15)-O(22)	1.415	1.422	C(4)-C(9)-O(10)	121.5	121.7
C(15)-H(36)	1.092	-	C(8)-C(9)-O(10)	114.8	117.4
C(16)-C(17)	1.542	1.513	C(1)-O(10)-C(9)	122.0	
C(16)-O(21)	1.440	1.416	C(2)-O(12)-C(14)	117.2	116.1
C(16)-H(37)	1.095	-	C(14)-O(13)-C(18)	115.5	116.3
C(17)-C(18)	1.536	1.542	O(12)-C(14)-O(13)	107.9	112.3
C(17)-O(20)	1.425	1.426	O(12)-C(14)-C(15)	106.1	105.3
C(17)-H(38)	1.095	-	O(12)-C(14)-H(35)	109.9	-
C(18)-C(19)	1.529	1.511	O(13)-C(14)-C(15)	111.3	112.9
C(18)-H(39)	1.093	-	O(13)-C(14)-H(35)	110.1	-
C(19)-H(40)	1.094	-	C(15)-C(14)-H(35)	111.4	-
C(19)-H(41)	1.089	-	C(14)-C(15)-C(16)	111.2	109.4
C(19)-H(42)	1.093	-	C(14)-C(15)-O(22)	112.1	107.7
O(20)-H(43)	0.967	-	C(14)-C(15)-H(36)	106.2	-
O(21)-H(44)	0.961	-	C(16)-C(15)-O(22)	110.6	110.6
O(22)-H(45)	0.967	-	C(16)-C(15)-H(36)	109.3	-
O(23)-H(46)	0.963	-	O(22)-C(15)-H(36)	107.2	-
O(24)-H(47)	0.963		C(15)-C(16)-C(17)	111.8	110.0
C(25)-C(26)	1.409	1.396	C(15)-C(16)-O(21)	106.0	113.2
C(25)-C(30)	1.404	1.398	C(15)-C(16)-H(37)	108.2	-
C(26)-C(27)	1.382	1.380	C(17)-C(16)-O(21)	112.4	107.8
C(26)-H(48)	1.083	-	C(17)-C(16)-H(37)	108.5	-
C(27)-C(28)	1.404	1.401	O(21)-C(16)-H(37)	109.9	-
C(27)-O(32)	1.378	1.358	C(16)-C(17)-C(18)	112.1	112.3
C(28)-C(29)	1.390	-	C(16)-C(17)-O(20)	108.8	110.6
C(28)-O(31)	1.358	1.359	C(16)-C(17)-H(38)	109.4	-
C(29)-O(30)	1.391	-	C(18)-C(17)-O(20)	109.6	109.9
C(29)-H(49)	1.083	-	C(18)-C(17)-H(38)	110.3	-
O(30)-H(50)	1.079	-	O(20)-C(17)-H(38)	106.5	-
O(31)-H(51)	0.966	-	O(13)-C(18)-C(17)	107.1	111.3
			O(13)-C(18)-C(19)	113.7	106.5
			O(13)-C(18)-H(39)	103.1	-
			C(17)-C(18)-C(19)	116.9	111.6
			C(17)-C(18)-H(39)	106.5	-
			C(19)-C(18)-H(39)	108.4	-
			C(18)-C(19)-H(40)	109.3	-
			C(18)-C(19)-H(41)	113.1	-
			C(18)-C(19)-H(42)	109.2	-
			H(40)-C(19)-H(41)	107.7	-
			H(40)-C(19)-H(42)	108.0	-
			H(41)-C(19)-H(42)	109.4	-
			C(17)-O(20)-H(43)	106.9	-
			C(16)-O(21)-H(44)	108.9	-
			C(15)-O(22)-H(45)	105.7	-
			C(7)-O(23)-H(46)	109.6	-
			C(5)-O(24)-H(47)	108.8	-
			C(1)-C(25)-C(26)	118.9	119.4
			C(1)-C(25)-C(30)	122.6	121.5
			C(26)-C(25)-C(30)	118.4	118.4
			C(25)-C(26)-C(27)	120.6	121.3
			C(25)-C(26)-H(48)	119.8	-
			C(27)-C(26)-C(28)	119.6	-
			C(26)-C(27)-C(28)	120.7	119.5
			C(26)-C(27)-O(32)	124.5	118.5
			C(28)-C(27)-O(32)	114.8	122.3
			C(27)-C(28)-C(29)	118.9	119.5
			C(27)-C(28)-O(31)	120.6	114.9
			C(29)-C(28)-O(31)	120.5	125.4
			C(28)-C(29)-C(30)	120.8	125.4
			C(28)-C(29)-H(49)	118.6	-
			C(30)-C(29)-H(49)	120.6	-
			C(25)-C(30)-C(29)	120.5	120.7
			C(25)-C(30)-H(50)	120.4	-
			C(29)-C(30)-H(50)	119.0	-
			C(28)-O(31)-H(51)	107.8	-
			C(27)-O(32)-H(52)	110.0	-

^a^Experimental values taken from ref [26].

### 3.2 Vibrational assignments

**Figure 2:**
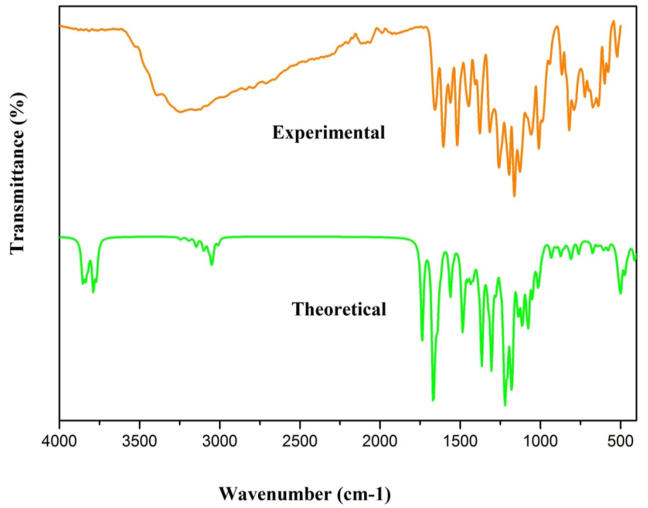
Calculated and experimental FT-IR spectra of quercitrin.

**Figure 3:**
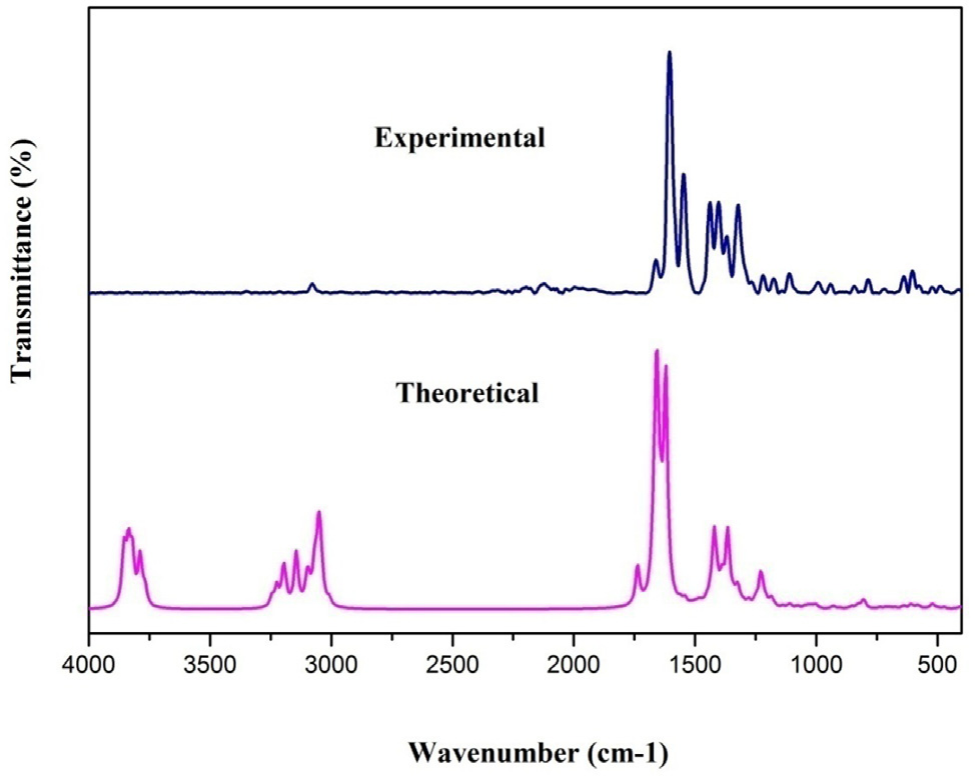
Calculated and Experimental FT-Raman spectra of Quercitrin.

The Quercitrin, which is found to be more stable, pertains to C1 point symmetry. The DFT method is used to estimate the vibrational spectroscopic studies with a basis set of 6–311++G(d, p), which has a scaling factor of 0.961 and its PED % is calculated [27]. Table 2 illustrates the theoretical and experimental vibrational variables of the quercitrin. The FT-IR and FT-Raman spectra of Quercitrin are shown in Figures 2 and 3, respectively.

**Table 2:** The experimental (FT-IR and FT-Raman) and theoretical vibrational frequencies using DFT for quercitrin.

Species	Experimental Wavenumbers(cm^−1^)		Theoretical wave number (cm^−1^)						Vibrational assignments
	FT-IR	FT-RAMAN	Unscaled	scaled	IR intensities		RAMAN Activities		
					Rel	Abs	Rel	Abs	
W(150)			3853	3703	55	13	119	14	OH stretching (92)
W(149)			3850	3700	68	16	97	12	OH stretching (100)
W(148)			3834	3684	94	22	199	24	OH stretching (92)
W(147)			3817	3668	58	14	187	22	OH stretching (100)
W(146)			3783	3636	146	34	181	22	OH stretching (100)
W(145)	3611		3764	3617	77	18	12	1	OH stretching (100)
W(144)			3763	3616	30	7	53	6	OH stretching (100)
W(143)	3122		3242	3115	8	2	37	4	CH stretching (99)
W(142)		3078	3221	3095	0	0	72	9	CH stretching (100)
W(141)			3194	3069	5	1	138	17	CH stretching (99)
W(140)			3185	3061	6	1	30	4	CH stretching (97)
W(139)			3143	3021	11	3	16	2	CH stretching (98)
W(138)			3139	3017	19	5	189	23	CH stretching (98)
W(137)			3097	2976	30	7	78	9	CH stretching (93)
W(136)			3086	2966	12	3	61	7	CH stretching (92)
W(135)			3064	2945	24	6	136	16	CH stretching (98)
W(134)			3046	2927	49	12	244	29	CH stretching (94)
W(133)			3039	2921	44	10	93	11	CH stretching (95)
W(132)			3036	2917	3	1	60	7	CH stretching (84)
W(131)	2816		3003	2886	21	5	26	3	CH stretching (97)
W(130)	1641	1660	1733	1665	324	76	145	17	OC stretching (85)
W(129)	1602	1604	1665	1600	424	100	239	29	CC stretching (62)
W(128)			1657	1593	31	7	29	3	CC stretching (47)
W(127)			1653	1588	129	31	787	94	CC stretching (26)
W(126)	1557		1635	1571	194	46	165	20	CC stretching (30)
W(125)	1517	1546	1617	1554	54	13	836	100	CC stretching (22)
W(124)			1558	1497	167	39	13	2	CC stretching (42)
W(123)			1536	1476	23	6	23	3	CC stretching (38)
W(122)	1445	1438	1512	1453	5	1	5	1	CC stretching (26)
W(121)			1494	1436	7	2	10	1	CC stretching (21)
W(120)	1419		1481	1423	248	58	14	2	CC stretching (41)
W(119)		1402	1469	1412	68	16	8	1	CC stretching (32)
W(118)	1387		1448	1392	67	16	15	2	OC stretching (10)
W(117)			1431	1375	46	11	24	3	OC stretching (56)
W(116)		1368	1428	1372	35	8	9	1	OC stretching (10)
W(115)			1417	1362	30	7	184	22	OC stretching (22)
W(114)			1415	1359	12	3	50	6	OC stretching (10)
W(113)			1412	1357	27	6	29	3	CC bending (20)
W(112)			1406	1351	16	4	48	6	OC stretching (30)
W(111)			1395	1341	5	1	2	0	OC stretching (10)
W(110)			1385	1331	21	5	81	10	CC stretching (10)
W(109)		1322	1382	1328	29	7	4	0	OC stretching (20)
W(108)	1315		1372	1318	31	7	12	1	OC stretching (52)
W(107)			1362	1309	349	82	257	31	OC stretching (36)
W(106)			1354	1301	4	1	16	2	OC stretching (66)
W(105)			1334	1282	24	6	28	3	CC stretching (22)
W(104)			1318	1267	90	21	46	6	CC stretching (13)
W(103)	1256		1316	1265	20	5	24	3	OC stretching (14)
W(102)			1300	1249	348	82	10	1	CC stretching (32)
W(101)			1293	1243	14	3	6	1	CC stretching (22)
W(100)			1274	1225	33	8	7	1	OC stretching (49)
W(99)			1273	1223	42	10	12	1	CCC bending (10)
W(98)	1205	1217	1267	1217	36	8	3	0	CCC bending (28)
W(97)			1243	1195	50	12	26	3	CCC bending (12)
W(96)		1175	1225	1177	111	26	113	14	CCO bending (11)
W(95)			1218	1170	318	75	26	3	HOC bending (56)
W(94)			1214	1166	33	8	2	0	HOC bending (63)
W(93)	1158		1205	1158	8	2	16	2	HOC bending (57)
W(92)			1199	1152	232	55	7	1	HOC bending (46)
W(91)			1182	1136	168	40	33	4	HOC bending (54)
W(90)			1172	1126	194	46	0	0	HOC bending (45)
W(89)	1120	1110	1168	1123	132	31	7	1	HOC bending (49)
W(88)			1136	1092	96	23	3	0	HCC bending (48)
W(87)			1134	1089	34	8	4	0	HCC bending (40)
W(86)			1129	1085	56	13	2	0	HCC bending (57)
W(85)	1067		1113	1070	114	27	3	0	HCC bending (33)
W(84)			1104	1061	116	27	10	1	HCC bending (23)
W(83)			1098	1056	17	4	4	0	HCO bending (69)
W(82)			1082	1039	91	21	3	0	HCO bending (23)
W(81)			1071	1029	61	14	2	0	HCO bending (40)
W(80)			1066	1025	138	32	5	1	HCC bending (44)
W(79)	1004	991	1046	1005	132	31	7	1	HCC bending (60)
W(78)			1028	988	18	4	10	1	HCH bending (59)
W(77)	970		1014	975	16	4	6	1	HCH bending (62)
W(76)			1009	969	105	25	5	1	HCH bending (59)
W(75)			997	958	44	10	14	2	OCC bending (15)
W(74)		940	982	943	15	3	1	0	CCO bending (22)
W(73)			930	894	45	11	7	1	CCC bending (25)
W(72)	865		918	882	15	4	5	1	CCO bending (13)
W(71)			894	859	15	4	3	0	CCC bending (12)
W(70)	832		867	833	50	12	1	0	CCC bending (20)
W(69)			850	817	14	3	7	1	CCC bending (16)
W(68)	807		843	810	4	1	2	0	CCO bending (14)
W(67)			824	792	6	1	6	1	CCC bending (16)
W(66)	787	785	819	787	3	1	9	1	OCC bending (37)
W(65)	774		808	777	34	8	2	0	OCC bending (44)
W(64)			798	767	35	8	28	3	COC bending (11)
W(63)			790	759	3	1	8	1	OCC bending (42)
W(62)	722		755	726	47	11	1	0	OCC bending (37)
W(61)			732	704	5	1	6	1	CCC bending (14)
W(60)	682		712	685	3	1	3	0	OCC bending (11)
W(59)			705	678	2	0	3	0	OCC bending (38)
W(58)			691	664	4	1	5	1	COC bending (15)
W(57)			673	647	4	1	4	0	OCC bending (18)
W(56)	624	638	666	640	42	10	2	0	OCO bending (20)
W(55)			639	614	13	3	5	1	OCC bending (12)
W(54)		602	633	608	6	2	7	1	OCC bending (51)
W(53)	592		619	595	8	2	1	0	OCC bending (10)
W(52)			606	582	17	4	15	2	OCC bending (20)
W(51)	572		596	573	17	4	2	0	OCC bending (40)
W(50)			586	563	2	0	6	1	OCC bending (35)
W(49)			573	551	22	5	9	1	HOCC torsion (58)
W(48)			571	548	8	2	2	0	HOCC torsion (78)
W(47)	526		549	528	3	1	2	0	HOCC torsion (75)
W(46)			534	513	7	2	1	0	HOCC torsion (89)
W(45)		487	516	496	28	7	18	2	HOCC torsion (78)
W(44)			503	483	79	19	2	0	HOCC torsion (88)
W(43)			490	471	102	24	2	0	HOCC torsion (83)
W(42)			490	471	23	5	1	0	HCCC torsion (75)
W(41)			469	450	51	12	4	0	HCCC torsion (67)
W(40)			468	450	20	5	2	0	HCCC torsion (74)
W(39)			466	448	19	5	1	0	HCCC torsion (79)
W(38)			448	430	28	7	1	0	HCCC torsion (82)
W(37)			430	413	6	2	2	0	HCOC torsion (33)
W(36)			409	393	42	10	2	0	HCOC torsion (28)
W(35)			403	388	15	4	3	0	HCCC torsion (50)
W(34)			391	376	17	4	5	1	HCCC torsion (43)
W(33)			373	359	27	6	2	0	HCCC torsion (49)
W(32)			359	345	15	4	3	0	CCCC torsion (36)
W(31)			345	332	4	1	1	0	CCCO torsion (36)
W(30)			343	330	108	25	4	0	CCCC torsion (51)
W(29)			337	324	11	3	0	0	CCOC torsion (52)
W(28)			324	312	2	0	1	0	CCCC torsion (29)
W(27)			312	300	3	1	0	0	CCCC torsion (37)
W(26)			301	289	11	3	1	0	CCCC torsion (37)
W(25)			296	285	1	0	3	0	COCC torsion (44)
W(24)			267	257	18	4	2	0	OCCC torsion (63)
W(23)			267	256	19	5	0	0	COCC torsion (58)
W(22)			256	246	25	6	3	0	OCOC torsion (81)
W(21)			250	241	16	4	1	0	COCO torsion (50)
W(20)			239	230	0	0	0	0	CCCO torsion
W(19)			233	224	6	1	1	0	CCOC torsion (14)
W(18)			221	213	21	5	2	0	CCCH out (67)
W(17)			219	210	31	7	4	0	CCCH out (31)
W(16)			217	208	71	17	2	0	CCCH out (26)
W(15)			205	197	14	3	2	0	OCCC out (43)
W(14)			199	192	4	1	1	0	OCCC out (28)
W(13)			181	174	8	2	0	0	OCCC out (37)
W(12)		162	172	165	1	0	1	0	OCCC out (10)
W(11)			137	131	0	0	3	0	OCCC out (11)
W(10)			114	109	2	1	0	0	OCCC out (21)
W(9)			93	89	0	0	0	0	OCCC out (36)
W(8)			92	88	3	1	0	0	OCCC out (58)
W(7)		75	78	75	0	0	1	0	CCOC out (22)
W(6)			61	58	0	0	2	0	COOC out (20)
W(5)			46	44	0	0	4	0	OCCC out (38)
W(4)			37	36	1	0	1	0	CCCC out (23)
W(3)			29	28	1	0	3	0	CCOC out (22)
W(2)			24	23	1	0	2	0	CCOC out (40)
W(1)			14	13	1	0	3	0	CCCC out (38)

1. Stretching, Bending, Torsion and Out.

2. Scaling Factor 0.961 for B3LYP6-311++G (d,p) [ref. 27].

3. Relative absorption intensity normalized with highest peak absorption equal to 100.

4. Relative Raman intensity normalized to 100.

#### 3.2.1 O–H vibrations

The O–H hydroxyl stretching vibrations are tremendously effective for intra or intermolecular hydrogen bonding in the quercitrin. K. Muthu et.al reported the hydroxyl stretching vibrations in the 3600-3400 cm^−1^ range [28]. The O–H stretching vibrations of the Quercitrin, predicted by theoretical calculation, were observed at 3703, 3700, 3684, 3636, 3617, and 3616 cm^−1^ with a 100 % PED contribution. It is supported by the experimental FT-IR spectra observed at 3611cm^−1^.

#### 3.2.2 C–H vibrations

The aromatic C–H stretching vibrations mostly appear in the range of 3100 to 3000 cm^−1^ [29]. Manjusha et.al reported the C–H stretching vibrations in the 2850-3000 cm^−1^ range [30]. In Quercitrin, C–H stretching vibrations occur from 3115 to 2886 cm^−1^. The experimental bands of FT-IR spectra were observed at 3122 and 2816 cm^−1^ and FT-Raman was observed at 3078 cm^−1^. The maximum PED contribution is 100%. Moreover, HCC bending bands are registered at 1067 and 1004 cm^−1^ in FT-IR and 991cm^−1^ in FT-Raman. Theoretically, HCH bending bands appear at 983,970 cm^−1^.

#### 3.2.3 C–O vibrations

The stretching vibrations of C–O and the carbonyl C=O group are commonly disclosed in the range of 1740–1660 cm^−1^ [31]. The C–O stretching bands of the Quercitrin are noticed theoretically at 1665, 1392, 1375, 1372, 1362, 1359,1351, 1341, 1328, 1318, 1309, 1301, 1265, and 1225 cm^−1^ for the 6–311 G (d,p) basis set. The experimental values noticed at 1452, 1242, 1175, 1128, and 1003 cm^−1^ for FT-IR spectra and values at 1177 and 1226 cm^−1^ for FT-Raman spectra are in great concurrence with the values predicted using theoretical calculations with the 6-311G++ (d,p) basis set. These values are supported by the average potential energy distribution value of 85%. The blended vibration positions at 774,722, 682, 592, and 572cm^−1^for FT-IR are assigned to the OCC bending vibration. The other COC bending vibrations are observed at 624 in FT-IR and 638 cm^−1^ in FT-Raman. The CCO bending vibrations are recorded at 865, 807 and 787cm^−1^in FT-IR and at 1175 and 940cm^−1^in FT-Raman.

#### 3.2.4 C–C vibrations

In carbon-carbon stretching vibrations, the bands between 1300–1000 cm^−1^ are designated as C–C ring stretching vibrations [32, 33]. The theoretical C–C stretching vibration for Quercitrin is reported at 1600, 1593, 1653, 1635, 1617, 1558, 1536, 1512, 1494, 1481, 1467, 1357, 1331, 1282, 1267, 1249, and 1243 cm^−1^. For the Quercitrin, the experimental CC stretching vibration values in FT-IR are observed at 1600, 1557, 1517, 1445, and 1419 cm-1, and the FT-Raman values are 1604, 1546, 1438, and 1402 cm-1.

### 3.3 Frontier molecular orbitals (FMO's)

**Figure 4:**
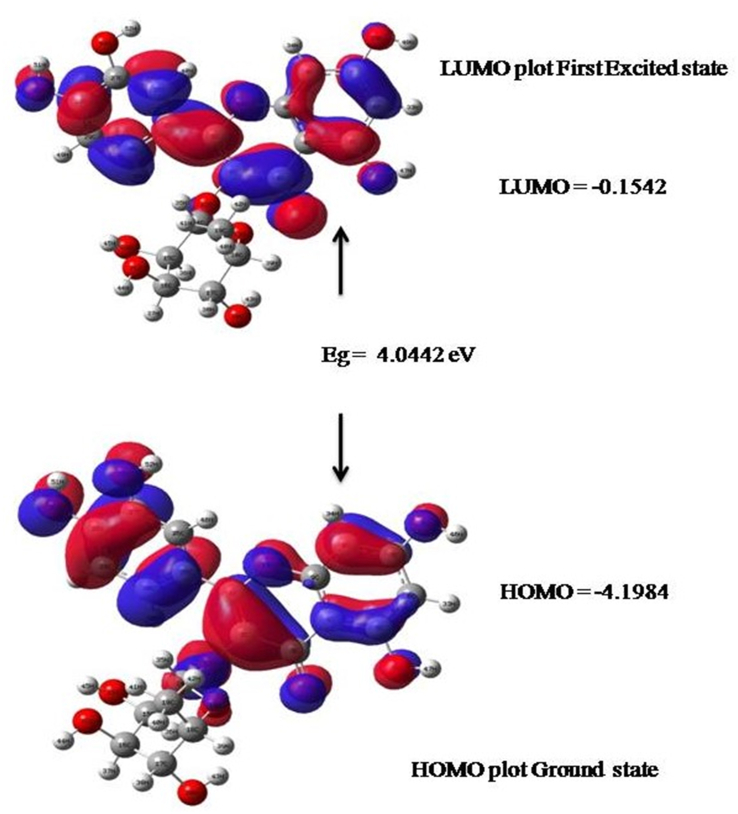
Frontier molecular orbital of the Quercitrin molecule.

FMOs assume a significant part during molecular interaction. The HOMO-LUMO energy gap and other FMO energies of the Quercitrin were plotted and represented in Figure 4. The FMOs fundamental descriptors are represented in Table 3 and Figure 4, [34, 35, 36]. The calculated HOMO energy value is -4.198eV, the LUMO value is -0.154 eV, and the bandgap energy value is 4.044 eV, for the quercitrin compound. It shows good charge transfer inside the molecule, and its biological activity is high [37]. The chemical softness of the quercitrin was found to be 0.247 eV. The low softness values of the quercitrin confirm the high stability and reactivity of the molecule. The chemical hardness of the quercitrin was computed to be 2.022 eV. The chemical potential and electrophilicity index values of quercitrin show that the molecule is a good bioactive drug.

**Table 3:** Global chemical reactivity descriptors of the Quercitrin.

Parameters (eV)	values
HOMO energy (E_HOMO_)	-4.1984
LUMO energy (E_LUMO_)	-0.1542
Energy gap (eV)	4.0442
Ionization potential(I)	4.1984
Electron affinity(A)	0.1542
Electronegativity (χ)	2.1763
Chemical potential (μ)	-2.1763
Chemical Hardness (η)	2.0221
Chemical softness (S)	0.2472
Electrophilicity (ω)	1.1711

### 3.4 Molecular electrostatic potential

**Figure 5:**
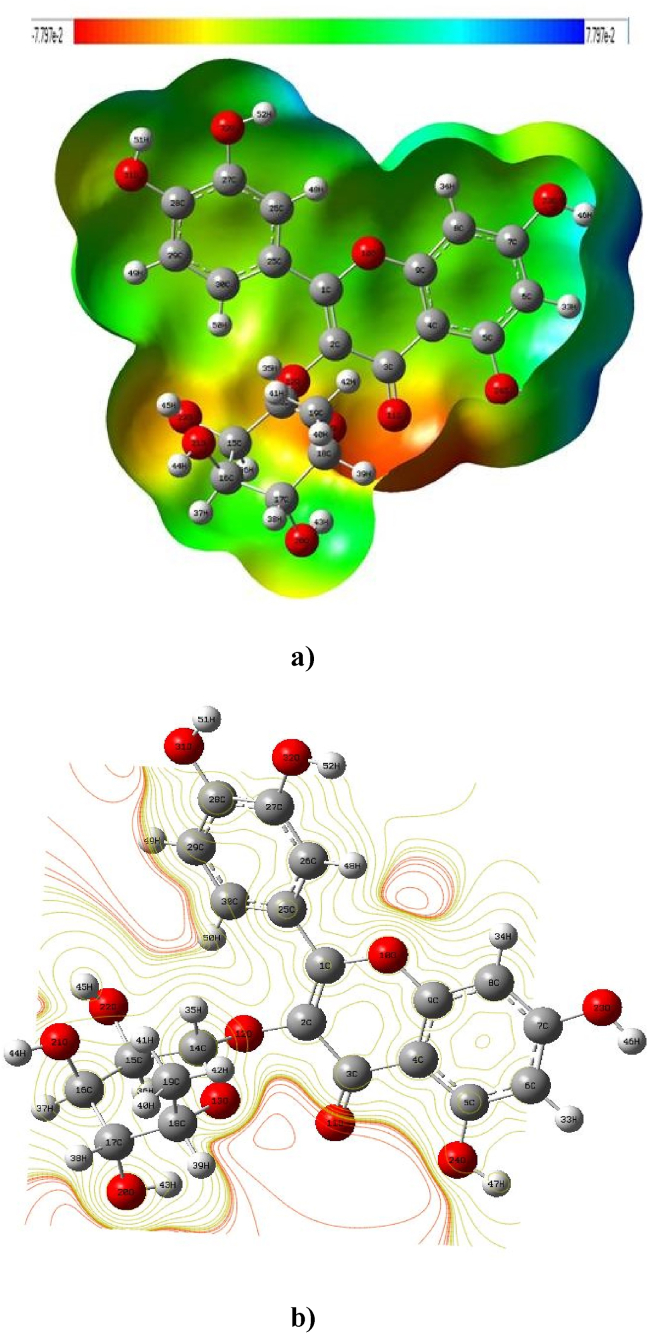
a) Molecular Electrostatic Potential surface map of Quercitrin and b) Contour map of electrostatic potential of the total density of the Quercitrin.

The molecular electrostatic potential surface Using the Gaussview 5.0 tool, for the quercitrin is depicted in Figure 5 (a) [38,39]. The MEP surface is exposed by various colors due to the increasing electron density in the order of red < orange < yellow < green < sky blue < blue. The 3D colour code MEP map ranges between-7.797 × 10^−2^ (red colour) and 7.797 × 10^−2^ (blue colour) for the Quercitrin, whereas the red color indicates the nucleophilic attack, which is the strongest attraction and the blue colour expresses electrophilic attack, which is the strongest repulsion. In the quercitrin, the negative region is more focused around the O(10), O(11), O(12), O(13), O(20), O(21), O(22), O(23), O(24), O(31), and O(32) atoms. Figure 5(b) depicts the contour map for Quercitrin, which clearly shows the density around various atoms.

### 3.5 Local reactivity descriptors

**Figure 6:**
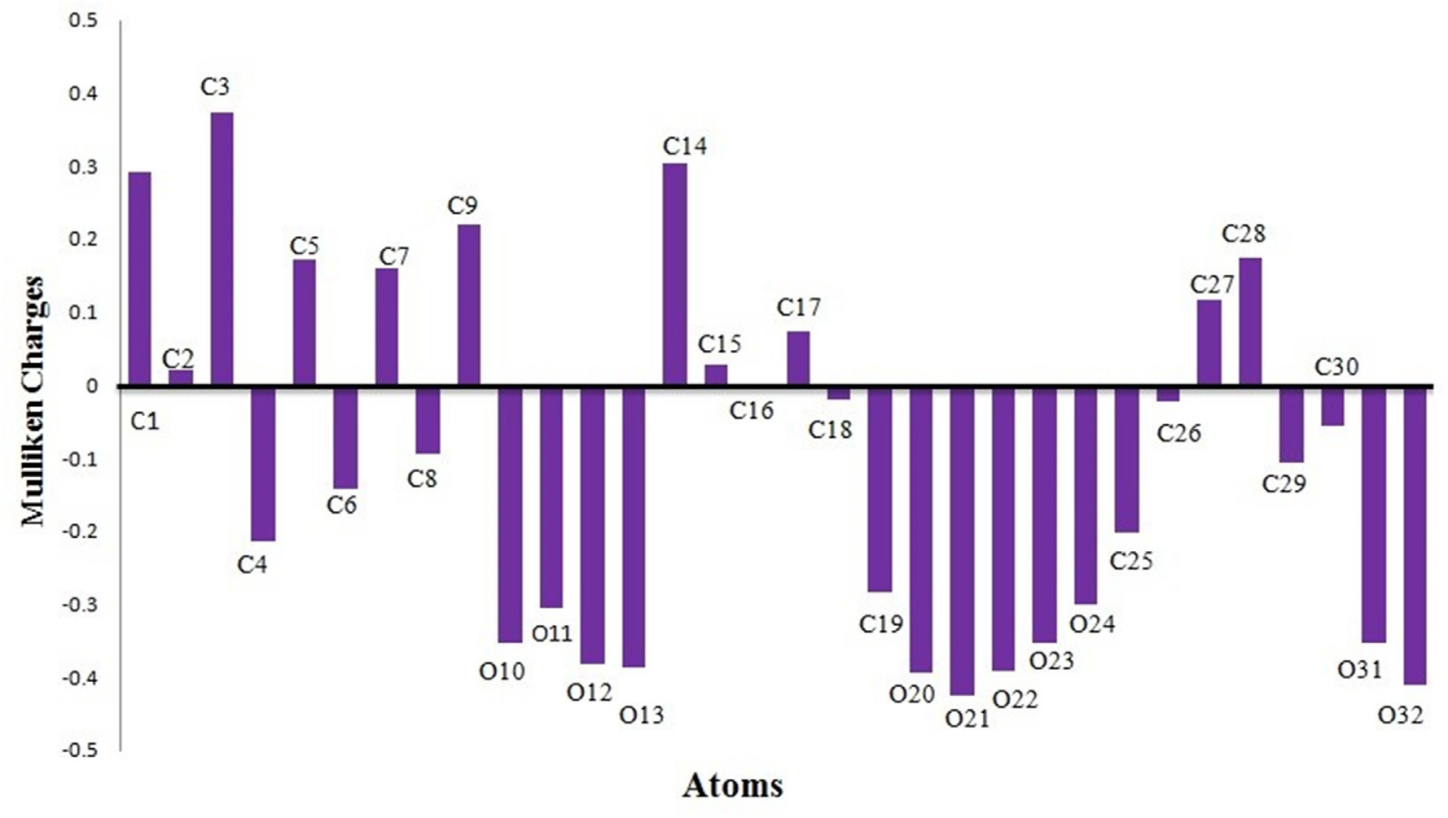
The histogram of calculated Mulliken charge of Quercitrin molecule expect hydrogen.

The Fukui function is an important aspect of developing a pharmaceutical product since it is used to identify the electron density based on local reactivity descriptors, which are used to predict the chemical reactivity of the molecule [40, 41, 42, 43]. The atoms may possess a positive or negative mulliken charge in accordance with the number of electrons surrounding them. Figure 6 depicts the histogram of the computed Mulliken charge of the Quercitrin without hydrogen atoms, as well as all the oxygen atoms that have a negative charge and carbon atoms C1 to C9, C14, C15, C17, C27, and C28 that have a positive charge, while the molecule's other carbon atoms have negative charges. C3 has the highest positive value (0.389) of all the carbon atoms, whereas the oxygen atom has the highest negative value (−0.433) and the results are reported in Table 4. The computed f_k_^+^ value shows the possible site for nucleophilic attack (due to positive value) and is in the order of C1, C2, C6, C8, O24, C27, C28, C30, O31, and O, and the f_k_^−^ value shows the possible site for electrophilic attack (due to negative value) and is in the order of C4, C7, C9, C18, and C25.

**Table 4:** Condensed Mulliken atomic charges, Fukui function f_k_ and descriptors (sf)_k_ and (ωf)_k_ values for Quercitrin.

Atom. No	Mulliken atomic charges			Fukui Function		Local softness		Electrophilicity index	
	q_N+1_	q_N_	q_N-1_	f_k_^+^	f_k_^-^	(sf)^+^	(sf)^-^	(ωf)^+^	(ωf)^-^
C1	0.318	0.291	0.240	0.027	0.051	0.007	0.013	0.032	0.060
C2	0.098	0.020	-0.016	0.078	0.036	0.019	0.009	0.091	0.042
C3	0.389	0.372	0.250	0.017	0.122	0.004	0.030	0.020	0.143
C4	-0.214	-0.214	-0.177	0.010	-0.037	0.010	-0.009	0.000	-0.044
C5	0.190	0.171	0.098	0.019	0.072	0.005	0.018	0.023	0.085
C6	-0.107	-0.142	-0.125	0.035	-0.017	0.009	-0.004	0.041	-0.020
C7	0.168	0.160	0.169	0.008	-0.009	0.002	-0.002	0.009	-0.011
C8	-0.053	-0.094	-0.117	0.040	0.024	0.010	0.006	0.047	0.028
C9	0.212	0.218	0.269	-0.007	-0.051	-0.002	-0.013	-0.008	-0.060
O10	-0.337	-0.354	-0.514	0.017	0.160	0.004	0.040	0.020	0.187
O11	-0.250	-0.305	-0.344	0.055	0.039	0.014	0.010	0.064	0.046
O12	-0.358	-0.383	-0.419	0.026	0.035	0.006	0.009	0.030	0.041
O13	-0.390	-0.387	-0.291	-0.003	-0.096	-0.001	-0.024	-0.003	-0.113
C14	0.288	0.303	0.264	-0.015	0.039	-0.004	0.010	-0.017	0.046
C15	0.023	0.028	0.093	-0.005	-0.065	-0.001	-0.016	-0.006	-0.077
C16	0.002	-0.001	0.010	0.003	-0.011	0.001	-0.003	0.003	-0.013
C17	0.070	0.074	0.062	-0.003	0.011	-0.001	0.003	-0.004	0.013
C18	-0.032	-0.019	0.050	-0.013	-0.069	-0.003	-0.017	-0.015	-0.081
C19	-0.283	-0.284	-0.323	0.001	0.039	0.000	0.010	0.001	0.046
O20	-0.376	-0.396	-0.394	0.019	-0.001	0.005	0.000	0.023	-0.001
O21	-0.433	-0.426	-0.407	-0.007	-0.019	-0.002	-0.005	-0.008	-0.022
O22	-0.392	-0.393	-0.397	0.000	0.004	0.000	0.001	0.000	0.005
O23	-0.327	-0.353	-0.423	0.027	0.069	0.007	0.017	0.031	0.081
O24	-0.279	-0.301	-0.379	0.022	0.079	0.005	0.019	0.026	0.092
C25	-0.188	-0.202	-0.156	0.015	-0.047	0.004	-0.012	0.017	-0.055
C26	0.011	-0.023	-0.082	0.034	0.059	0.008	0.014	0.039	0.069
C27	0.149	0.117	0.145	0.032	-0.029	0.008	-0.007	0.038	-0.033
C28	0.206	0.173	0.155	0.033	0.018	0.008	0.004	0.039	0.021
C29	-0.082	-0.107	-0.134	0.025	0.027	0.006	0.007	0.030	0.032
C30	-0.020	-0.056	-0.101	0.036	0.045	0.009	0.011	0.042	0.053
O31	-0.286	-0.354	-0.431	0.067	0.077	0.017	0.019	0.079	0.091
O32	-0.374	-0.412	-0.451	0.038	0.038	0.009	0.009	0.045	0.045

### 3.6 ADMET, drug-likeness properties and bioactivity score

Lipinski's rule of five [44, 45] and ADMET prediction [46] are both applied to assess the bioavailability of bulk material in drug discovery and development. In this study all the molecular characteristics of the Quercitrin were examined using the Molinspiration cheminformatics tool. Since the H-bond acceptor range is 11 (>10) and the H-bond donor range is 7 (>5) and TPSA 190 (>140), Quercitrin does not obey Lipinski's rule of five, as shown in Table 5 [47]. From ADMET, it is found that the compound quercitrin is orally available to humans, and the results are represented in Table 6. The AMES test's toxicity demonstrates that it is a non-carcinogenic and non-mutagenic molecule. The title compound was seen as a non-inhibitor for hERG (human ether -a-go-go gene), which proposes that it does not hinder any potassium channels.

**Table 5:** Prediction of drug-likeness properties for Quercitrin.

Descriptors	Properties
Hydrogen bond donor (HBD)	7
Hydrogen bond acceptor (HBA)	11
Partition coefficient (MilogP)	0.64
Molecular weight (MW)	448.34
Topological polar surface area (TPSA) (Å^2^)	190.2
Number of atoms	52
Number of rotatable bonds	3

**Table 6:** Prediction of ADMET profiles of Quercitrin.

A	B	C	D	E	F	G	H	I	J
0.0393	Non	52.709	76.443	-4.573	Suitable	Qualified	Mutagen	Violated	Non-inhibitor

A:ADMET_BBB.

B: P-glycoprotein inhibitor.

C: Human intestinal absorption (HIA+, %).

D: Plasma protein binding (PPB, %).

E: ADMET_SK logP.

F: Lipinski's rule.

G:CMC-like rule.

H:Ames_test

I: Lead-like rule.

J: hERG- I&II inhibitor.

Molinspiration is a web server tool utilized to predict the bioactivity score of the Quercitrin against regular human targets. The values are given in Table 7 which shows it as a moderately active compound. Bioactive scores for kinase protein, nuclear receptor ligands, and enzyme inhibitors were 0.08, 0.17, and 0.37, respectively. The projected values for GPCR, ion channel modulators, and protease inhibitors are -0.01, -0.08, and -0.06, respectively: These expected values are relatively active. Based on these findings, we can conclude that quercitrin has the potential to be used as an anti-cancer drug in the future.

**Table 7:** Bioactivity score of Quercitrin.

Compound	GPCR	Ion channel modulator	Kinase inhibitor	Nuclear receptor ligand	Protease inhibitor	Enzyme inhibitor
Quercitrin	-0.01	-0.08	0.08	0.17	-0.06	0.37

### 3.7 Molecular docking study

**Figure 7:**
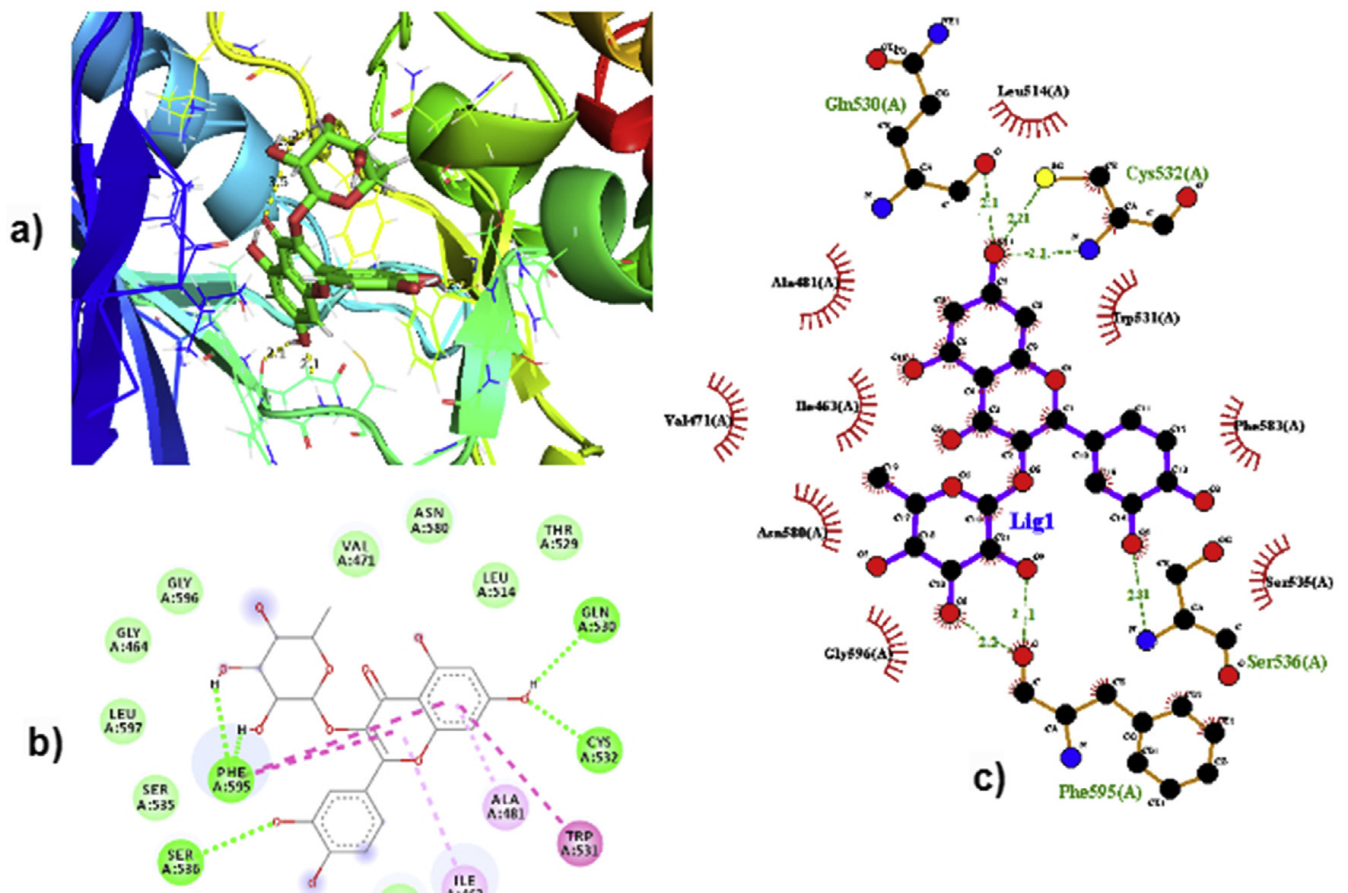
a) The position of the ligand Quercitrin in the binding cavity of the B-RAF Protein b) 2D view of distance for hydrogen bond interaction of Quercitrin with amino acid residues of B-RAF Protein and c) The ligplot showing intermolecular interaction of Quercitrin molecule in the active site of B-RAF Protein.

The optimized Quercitrin structure is converted to PDB format and docked with the B-RAF protein (RCSB with PDB ID 6B8U) [48]. The resolution of the corresponding protein has a lower value of 2.68Å so that we can get an optimum structure for the B-RAF protein. The active site of the B-RAF protein comprises the residues Glu 501, Cys 532, Asp 594, and Phe 595; respective binding energy values are shown in Table 8. The amino acids Phe 595, Gln 530, Cys 532, and Ser 536 of B-RAF protein forms strong conventional hydrogen bond interactions with atoms H(44), H(45), H(46), and H(51) of the Quercitrin with a distance of 2.2, 2.1, 2.3, and 2.2Å, respectively Figure 7 (a). The residues Trp 531, Ala 481, and Ile 463 of B-RAF protein form pi-pi stacked interaction with Quercitrin which is shown in Figure 7 (b). Figure 7 (c) represents the Quercitrin docked into the binding cavity (2D view, Ligplot) of the B-RAF protein. The compound which has numerically higher binding energy is found to possess increased binding affinity towards target protein [49]. This result reveals that quercitrin interacts well with the B-RAF kinase protein and fits well into the binding cavity of the B-RAF target protein. The binding affinity of the compound Quercitrin, which has a -7.14 kcal/mol using AutoDock, was validated with the Glide XP score in Schrodinger software (−8.01 kcal/mol). In addition, the binding score of Imidazopyridinyl benzamide bound with B-RAF protein was also calculated using AutoDock (−5.21 kcal/mol) and Schrodinger's Glide XP (−6.41). From these docking results, it is found that quercitrin has a numerically higher binding energy and is found to be a potent anti-cancer agent than Imidazopyridinyl benzamide.

**Table 8:** Molecular docking results and Hydrogen bonding distances between B-RAF protein and inhibitors such as Quercitrin and Imidazopyridinyl benzamide.

Compounds	Bonded residues	Bond distance (Å)	Binding energy Kcal/mol		Reference RMSD (Å)
			Autodock	Schrodinger maestro xpGlide	
Quercitrin	Phe 595 Ser 536 Gln 530 Cys 532	2.2 2.2 2.1 2.0	-7.14	-8.01	1.816
Imidazopyridinyl benzamide	Phe 595 Asp 594 Glu 501 Cys 532	3.1 2.9 2.8 2.5	-5.21	-6.41	1.780

### 3.8 Molecular dynamics simulations

#### 3.8.1 Analysis of structural stability, fluctuations and compactness of the protein

**Figure 8:**
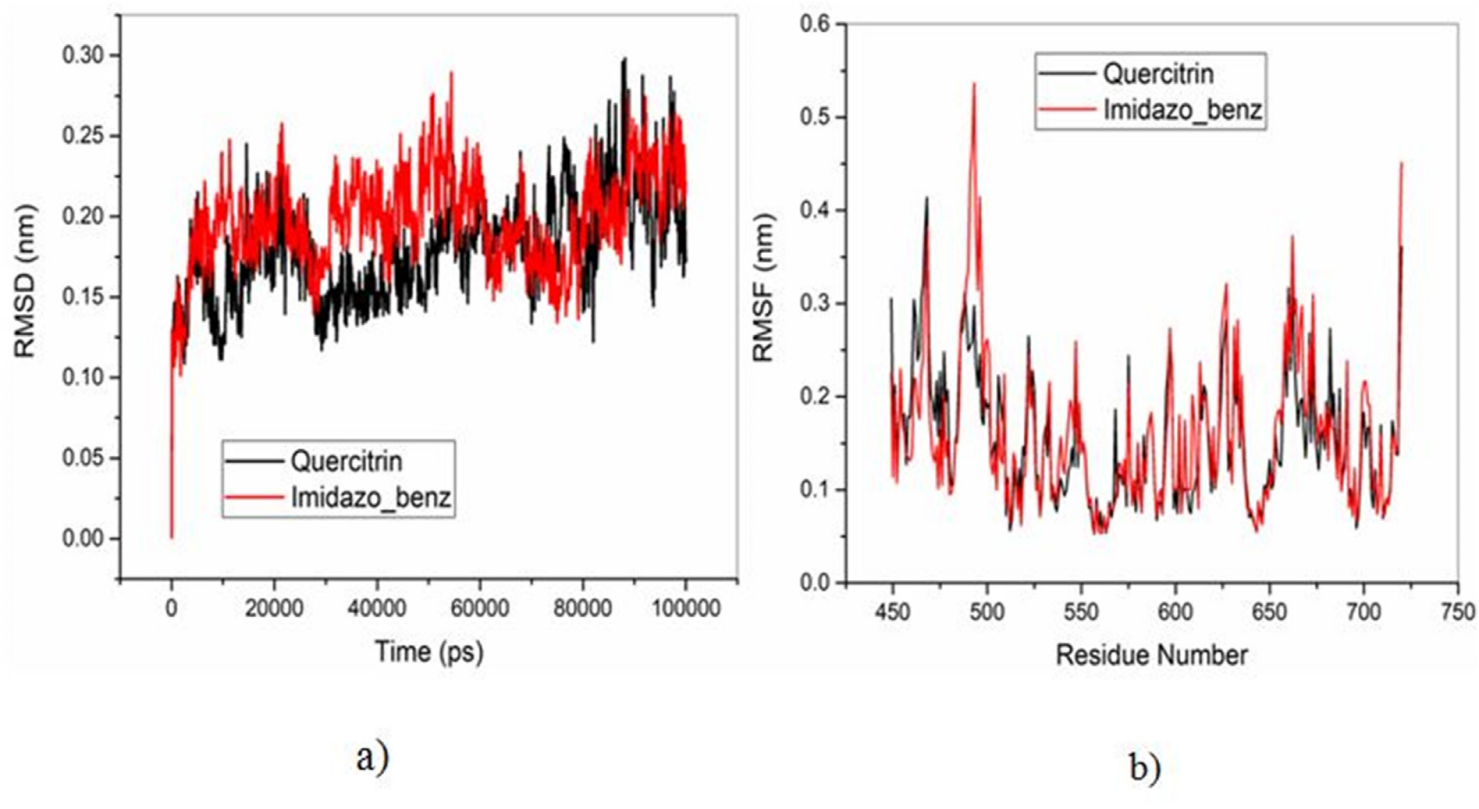
a) Root Mean Square Deviation (RMSD) and b) Root Mean Square Fluctuation (RMSF) of B-RAF protein bounded to Quercitrin and Imidazopyridinyl benzamide inhibitors.

**Figure 9:**
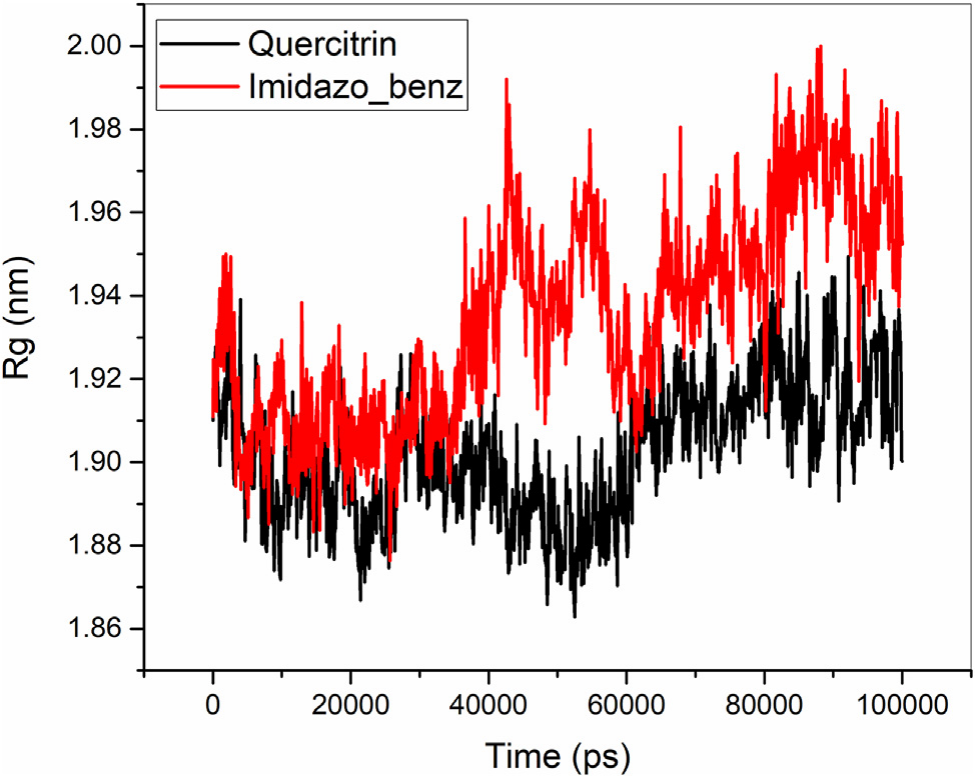
Radius of gyration (Rg) of the B-RAF protein bounded to Quercitrin and Imidazopyridinyl benzamide inhibitors.

The structural stability of the Quercitrin and Imidazopyridinyl benzamide inhibitors bound to B-RAF protein was performed and compared using the Root Mean Square Deviation (RMSD) as shown in Figure 8 (a). For the last 20 ns, the Quercitrin has maintained RMSD value of around 0.20 nm. Though both the inhibitors maintain good stability of the protein, quercitrin is found to possess a low RMSD value and has shown higher stability of the protein in comparison with the Imidazopyridinyl benzamide inhibitor. The fluctuations of amino acid residues of B-RAF protein bound to Quercitrin and Imidazopyridinyl benzamide inhibitors were carried out using Root Mean Square Fluctuations (RMSF) analysis as shown in Figure 8 (b). The residue at position 493 of the Imidazopyridinyl benzamide bound B-RAF protein shows higher fluctuations at 0.53 nm, whereas the Quercitrin bound protein has 0.29 nm. On comparing the fluctuations of the two inhibitors, the Quercitrin bounded protein has low fluctuations and shows higher stability of the protein structure. The compactness of the protein can be identified from the analysis of the radius of gyration (Rg). From Figure 9, it is clearly known that the Quercitrin bounded protein has a lower value of Rg value than the Imidazopyridinyl benzamide bounded B-RAF protein. The Rg value of the Quercitrin-bound protein remained at 1.91 nm for the final 40 ns of MD simulation, whereas the Rg value of the Imidazopyridinyl benzamide-bound B-RAF protein ended at 1.96 nm.

#### 3.8.2 Analysis of hydrogen bonding and interaction energy analysis

**Figure 10:**
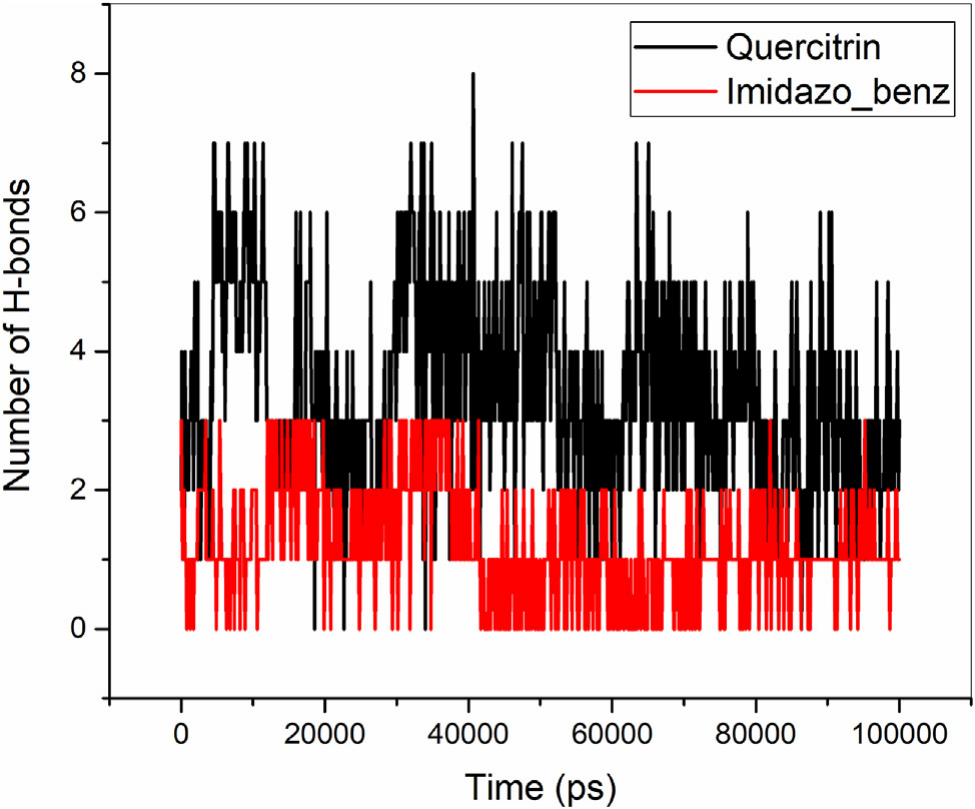
Hydrogen bonding interactions of Quercitrin and Imidazopyridinyl benzamide with B-RAF protein.

**Figure 11:**
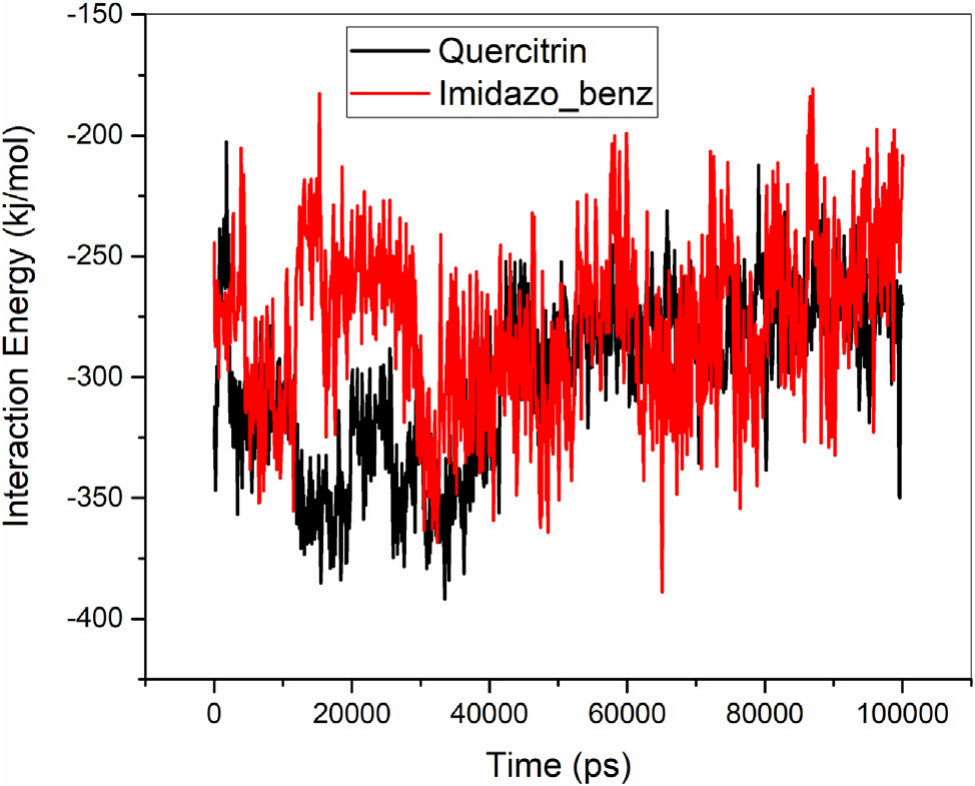
Total interaction energy between the inhibitors (Quercitrin and Imidazopyridinyl benzamide) and B-RAF protein.

The hydrogen bonds between the protein and ligands (Quercitrin and Imidazopyridinyl benzamide) were monitored throughout the MD simulation of 100 ns as shown in Figure 10. From Figure 10, it is evident that the Quercitrin has maintained 3 to 4 hydrogen bonds throughout the MD simulation, whereas the Imidazopyridinyl benzamide maintains 2 to 1 hydrogen bond. The total interaction energy, which is the sum of electrostatic and van der Waals (vdW) interactions, between the B-RAF protein and ligands (Quercitrin and Imidazopyridinyl benzamide), is shown in Figure 11 and Table 9. According to Table 9, quercitrin has a numerically higher interaction energy of 299.59 kj/mol (−71.60 kcal/mol) than Imidazopyridinyl benzamide, which has a numerically lower interaction energy of 269.39 kj/mol (−64.38 kcal/mol). For the two ligands, the vdW (−223.33 kj/mol (−53.37 kcal/mol) for Quercitrin and -169.63 kj/mol (−40.54 kcal/mol) for Imidazopyridinyl benzamide) interaction is found to be higher than electrostatic (−76.24 kj/mol (−18.22 kcal/mol) for Quercitrin and -99.76 kj/mol (−23.84 kcal/mol) for Imidazopyridinyl benzamide)) interactions. From this analysis, it is clearly known that quercitrin has higher affinity and is more bound to B-RAF protein than Imidazopyridinyl benzamide inhibitors.

**Table 9:** Total interaction energy of Quercitrin and Imidazopyridinyl benzamide inhibitors bounded to B-RAF protein. Energies are in kj/mol.

S. No.	Compounds	Electrostatic	vdW	Total interaction energy
1	Quercitrin	-99.76	-163.63	-269.39
2	Imidazopyridinyl benzamide	-76.24	-223.33	-299.59

## 4 Conclusion

The Quercitrin was structurally drawn and optimised to its lowest energy conformation, and bond length and bond angles were calculated and compared to experimental XRD data. The experimental and observed results of the Quercitrin compound's vibrational spectra (those based on PED %) were compared. The charge transfer inside the molecule is clearly described by the HOMO-LUMO, MEP, and Mulliken analyses. The molecular docking calculations were validated with two docking tools, AutoDock and Schrodinger's Glide XP. The binding affinity of the Quercitrin compound has a numerically higher binding affinity -7.14 kcal/mol (AutoDock) and -8.01 kcal/mol (Glide XP score in Schrodinger software) than the Imidazopyridinyl benzamide inhibitor.

In addition, MD simulations of protein-ligand complexes were monitored for 100 ns, from which the RMSD, RMSF, Rg, H-bonds, and interaction energy calculations were executed. Quercitrin is found to have a low RMSD value and has shown higher stability of the protein compared to Imidazopyridinyl benzamide inhibitor. From RMSF analysis, the Quercitrin bounded protein has low fluctuations and shows higher stability of the protein structure. Throughout the 100 ns of MD simulation, Quercitrin maintained 3 to 4 hydrogen bonds, whereas Imidazopyridinyl benzamide maintained 2 to 1 hydrogen bond. From these investigations, it is identified that the compound quercitrin has maintained good structural stability, compactness, higher Hydrogen bonds, and interaction energies than the Imidazopyridinyl benzamide inhibitors. Finally, it is concluded that quercitrin bounds well in the binding domain of B-RAF kinase protein.

## Declarations

### Author contribution statement

Govindammal M: Conceived and designed the experiments; Performed the experiments; Analyzed and interpreted the data; Contributed reagents, materials, analysis tools or data; Wrote the paper.

Kannan S: Performed the experiments; Analyzed and interpreted the data; Contributed reagents, materials, analysis tools or data.

Srinivasan P: Contributed reagents, materials, analysis tools or data.

Prasath M: Conceived and designed the experiments; Analyzed and interpreted the data; Wrote the paper.

### Funding statement

This research did not receive any specific grant from funding agencies in the public, commercial, or not-for-profit sectors.

### Data availability statement

Data included in article/supplementary material/referenced in article.

### Declaration of interests statement

The authors declare no conflict of interest.

### Additional information

No additional information is available for this paper.
